# Autoencoder-Ensemble-Based Unsupervised Selection of Production-Relevant Variables for Context-Aware Fault Diagnosis

**DOI:** 10.3390/s22218259

**Published:** 2022-10-28

**Authors:** Lukas Kaupp, Bernhard Humm, Kawa Nazemi, Stephan Simons

**Affiliations:** 1 Faculty of Computer Science, Darmstadt University of Applied Sciences, Haardtring 100, 64295 Darmstadt, Germany; 2 Research Group Human-Computer Interaction and Visual Analytics, Faculty of Media, Darmstadt University of Applied Sciences, Haardtring 100, 64295 Darmstadt, Germany; 3 Faculty of Electrical Engineering and Information Technology, Darmstadt University of Applied Sciences, Haardtring 100, 64295 Darmstadt, Germany

**Keywords:** context-aware diagnosis, outlier detection, cyber-physical systems, pre-training variable selection, autoencoder ensemble, production-relevant variables, OPC UA, Industry 4.0

## Abstract

Smart factories are complex; with the increased complexity of employed cyber-physical systems, the complexity evolves further. Cyber-physical systems produce high amounts of data that are hard to capture and challenging to analyze. Real-time recording of all data is not possible due to limited network capabilities. Limited network capabilities are the reason for a chain of faults introduced via active surveillance during fault diagnosis. These introduced faults may slow down production or lead to an outage of the production line. Here, we present a novel approach to automatically select production-relevant shop floor parameters to decrease the number of surveyed variables and, at the same time, maintain quality in fault diagnosis without overloading the network. We were able to achieve higher throughput, mitigate communication losses and prevent the disruption of factory instructions. Our approach uses an autoencoder ensemble via minority voting to differentiate between normal—always on—variables and production variables that may yield a higher entropy. Our approach has been tested in a production-equal smart factory and was cross-validated by a domain expert.

## 1. Introduction

Smart factories with their cyber-physical systems (CPSs) form a complex, highly integrated and interconnected environment. Each CPS produces a vast amount of data transmitted via various protocols. Such large amounts of data are hard to analyze because of the high number of variables that need to be surveyed and reasoned over. If a fault appears, the analyst searches for production-relevant variables that may be used to explain the fault based on domain knowledge and the faulty situation (context). This context is becoming more important in the fault diagnosis process [1].

For this reason, we proposed a Visual Analytics (VA) model called TAOISM (contex**t**-**a**ware diagn**o**sis **i**n **s**mart **m**anufacturing [2,3]). The TAOISM model reflects the context-aware fault diagnosis process in a smart factory. The context is mathematically defined in [2]. The integration on all stages of the fault diagnosis is also described in [2]. Context influences data retrieval, pre-processing and model building. It enables new ways for visualizations and knowledge generation involving analysts. Moreover, context facilitation may mitigate the increased complexity and help speed up problem solving in a rapidly changing environment under the Industry 4.0 paradigm. Along with new interaction possibilities, visualizations and models, we were able to show that context can be used in graph neural networks to reduce training time while maintaining a small reconstruction error for improved predictions and outlier detection [3]. Context-aware diagnosis with the TAOISM model is a part of Cognitive Manufacturing (CM). CM describes systems that can reason in several ways using a large quantity of data and respond robustly to unexpected changes [4]. In this manner, the TAOISM model may help in explaining a faulty situation with additional contextual information.

This work presents an unsupervised approach to extract production-relevant variables automatically without domain knowledge. This assists the analyst, e.g., during fault diagnosis. Moreover, maintenance cycles and live surveillance can benefit from a focused view on an otherwise massive number of surveyed variables. The publication considers the OPC UA standard as the underlying protocol for variable extraction, but the presented approach is also applicable for any type of variable extraction. The de facto standard in communication, OPC UA, tightly couples machinery with their hardware and software components. Each CPS has a corresponding OPC UA model, which keeps all information about the machinery. An OPC UA model consists of multiple stacked modules that all have several variables. An individual OPC UA model for specific machinery can consist of thousands of variables. A production line encompasses multiple models. Subscribing to all variables can lead to issues according to the available network bandwidth. Problems range from slowing down production to faulty products if a command is lost due to network latency. Security issues may also emerge if the networks are not strictly separated. Even if the network capacities are sufficient for transmitting all data, the hardware for handling all subscriptions may be limited (e.g., the OPC UA server used can only track 1000 messages at a time). Therefore, technical limitations hinder an analysis or live surveillance that benefits from a focused surveillance of certain production-relevant variables.

For those reasons, a solution is needed to focus on production-relevant variables within all available OPC UA models to obtain the maximum information from the available network capacities without interfering with the production process. For this, we need to specify the network’s capacity and describe the possible quota of information lost during the surveillance. Here, we present an automatic autoencoder (AE)-driven approach to detect production-relevant OPC UA variables based on the quantified information loss. All extracted variables can be surveyed in real-time without losing any subscribed data to network latency and bandwidth. The method focuses on variables (software or hardware components) that are active during production. The central hypothesis behind the approach is that active (production-relevant) variables are more likely involved in faulty situations and should be surveyed in real time. Therefore, we identify the production-relevant variables during production and order them by their activity level and extract a subset of the most active variables that can be surveyed in real time regarding the quantified information loss. The AE-based approach learns the behavior of a non-producing smart factory to distinguish between always-on variables (noise) and production activity. However, not all active variables are worthwhile to analyze, as production data can also have large text messages that are hard to interpret and retain minor information. Therefore, production data also need to be pre-filtered. The presented method covers these cases to retrieve a valuable subset of surveyable variables. Currently, AEs are common outlier detection techniques for distinguishing between normal and abnormal behavior over a set threshold. We found a way to describe the distinction between always-on and production variables as an outlier detection problem. Therefore, the term outlier has a specific meaning in our method. An outlier in this article is an active variable compared against the learned noise of a smart factory’s non-producing or production activity and not a production error.

Our approach has been tested against the production-equal smart factory [5,6] at Darmstadt University of Applied Sciences (DUAS). The smart factory produces electric relays identical to those found in wind turbines. The smart factory consists of high-bay storage (with a three-axis robot), a six-axis robot assembly station, a press and an optical and weight inspection, electrical inspection and manual inspection bay. All stations are interconnected via a monorail shuttle system with two shuttles. Each station encompasses a corresponding state-of-the-art OPC UA model that holds all information about different hardware modules (e.g., sensors) and software modules (e.g., production software) of the CPS.

We qualitatively evaluated the method and the variable subset by comparing the data pre-filtered and post-filtered by variable-frequency and OPC UA hierarchy representation. We can show that the technique learned an internal representation of the OPC UA hierarchy and selects production-relevant variables that have a chance—verified by a domain expert—to detect the contextual faults proposed in our previous work [6]. Moreover, we show that our approach is robust against over-represented OPC UA variables in the fed data. Additionally, the selection may provide a hint about CPS participation in the production process and may be used for reasoning in CPS fault participation in the future. By reducing variables, we were able to gain an additional 60% throughput of information without overloading the network. This increase in throughput is beneficial for a subsequent fault diagnosis, maintenance or live surveillance.

Our contribution is three-fold. (1) We develop a novel unsupervised approach to select production-relevant OPC UA variables without any employed domain knowledge. (2) We provide a novel voting strategy to facilitate minority voting in AE ensembles to detect outliers. (3) We detail the TAOISM model with an intelligent data selection strategy to support the context-aware diagnosis.

The remainder is structured as follows. Section 2 (Background) introduces the TAOISM model shortly and guides through the context-aware diagnosis. Section 3 (Related Work) provides an overview of today’s neural network-based outlier detection techniques in CPSs, ensemble methods, and methods to reduce training data, similarly to the proposed approach. Next, we explain the method in Section 4, the general idea, the used techniques, and the ensemble method with minority voting. Furthermore, Section 5 provides an overview of the smart factory, the data types, and the conversion strategy. Next, we discuss our results (Section 5.3). Finally, in Section 6, we conclude our work and pinpoint future directions.

## 2. Background: Context-Aware Diagnosis

The risen complexity in smart factories urges for novel fault diagnosis techniques. Here, we propose a methodology of context-aware diagnosis, where context is used to mitigate complexity in the diagnosis process in every stage of the analytical process. We released the TAOISM model [2,3] to integrate the context in the diagnosis process and scope discussions about different parts of the process. The model should be seen as open and living and as a guideline for future research affected throughout context-aware diagnosis processes.

**Figure 1:**
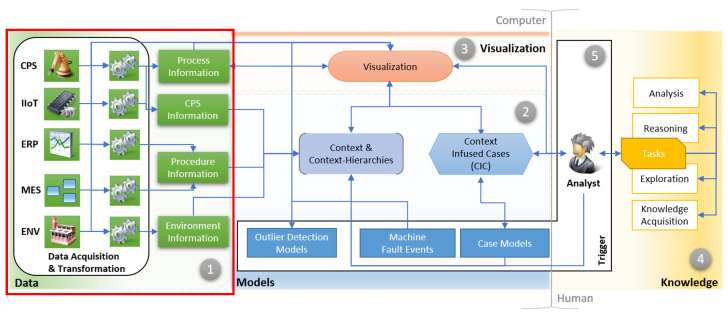
The TAOISM model [3]. The model reflects the context-aware diagnosis in smart manufacturing. The four main areas are Data (1), Models (2), Visualization (3) and Knowledge (4) and they align with the general VA model by Keim et al. [7], extended with a new meta-layer (Trigger, 5). The contribution adds to the model’s data acquisition (red squared). Adapted with permission from [3], “Context-Aware Diagnosis in Smart Manufacturing: TAOISM, An Industry 4.0-Ready Visual Analytics Model”; published by Springer Nature, 2022.

Context is a “situation within which something exists or happens, and that can help to explain it”. This is a vague definition, so we made an initial attempt to define the context of the current production situation mathematically [2,3]. The context is a window (or multiple windows) around an outlying timestamp over all available information sources [2] that may encompass other contexts in so-called context hierarchies [3]. Therefore, a context is a subset of information in which symptoms of unusual behavior during production are preserved. Here, we specify symptoms or outliers as a deviation from regular production activity. Moreover, the definition of context enables the integration in the pre-processing or the transformation step. One example is the integration in mathematical models (e.g., neural networks) or enabling novel navigation and visualization concepts. The last step in context-aware diagnosis is to mitigate complexity by guidance. An excerpt of information is shown to the analyst containing the outlying and surrounding information to reason the fault. This guidance allows for a quick start in fault exploration and may reduce the diagnosis process. The process is shown in Figure 1.

First, the different information sources produce data in the smart factory, e.g., CPSs, industrial internet of things (IIoT), enterprise resource planning (ERP), the manufacturing execution system (MES) or the environment (ENV). Next, different information sources are gathered in parallel. All enumerations in the model are just examples and should not be taken as exhaustive. Multiple technologies and protocols are involved in data acquisition, e.g., ProfiNet, OPC UA, serial and ethernet connections. Furthermore, there exist other methods, such as log surveillance, for recording even more data. In this article, we focus on a subset of all possible information sources, namely, the ones that are available via OPC UA—CPS, IIoT and environment information. All information must be transformed for beneficial use in mathematical models or NNs. We already released several strategies to transform and enrich data, from supervised [8,9] to unsupervised approaches [3,10]. A conversion challenge exists to convert all events and their values to a numerical format with a uniform distribution. Features have to be equally weighted (e.g., the distance between values is small and not large or extremely small numbers). Next, unsupervised techniques such as AEs are trained on online or offline data that serve as outlier detection models. Here, context can be facilitated in context hierarchies to learn, e.g., graph NNs, for faster training and improved context-based prediction [3]. Machine fault events represent machinery rule sets defined by the manufacturer that detect already-known fault cases (e.g., interruption of a safety sensor). Case models are either handcrafted or generated rules that cover different fault cases. Case models can be filled initially with already documented fault cases. Context-infused cases (CIC) are context annotated cases—already reasoned by a domain expert. Those cases are created after the context or context hierarchies are presented to the expert, and the expert connect the outlying values, e.g., over a custom ruleset together. These CIC cases, e.g., utilize multiple outlier detectors and rules to form a validated error detection model. Moreover, the expert can attach historical information and explanations to models. Context hierarchies in visualizations may be used to, e.g., alter the number of visual graphs, highlight the same information in different graphs or annotate specific values to various process steps. The first draft of visualizations can be found in [2,3]. Every expert has different daily tasks that should be supported, such as knowledge acquisition, exploration, analysis and reasoning.

Our previous work focuses on different aspects of the TAOISM model [2,3], such as supervised [8,9] or unsupervised models [3,10], visualization theory [11] (in German) or data [6]. There are still faults in a smart factory that are yet uncovered. These contextual faults emerge via the high level of automation focused on by the Industry 4.0 paradigm. We published the first set of contextual faults in a production-equal smart factory for the scientific community [6]. By definition, a contextual fault needs the current production situation or context to be reasoned. Therefore, this type of fault benefits from a context-aware diagnosis.

In this study, we tackle another open end in the context-aware diagnosis that exists throughout smart factories: the limitation of bandwidth, transfer capabilities and available information. Therefore, this publication details the data acquisition of the TAOISM model further. Most publications (Section 3) ignore the challenge entirely and start right after the data acquisition, on already recorded datasets or artificial data. However, a focused separation of available information, in association with the reduced network load, enables real-time diagnosis during production without interference. Therefore, more context-related information can be transferred, which may also benefit the downstream context-aware diagnosis process.

## 3. Related Work

Our approach downsizes the global OPC UA hierarchy to the appropriate size of production-relevant variables. The found subset is surveyable without production interference. Most publications spare the data acquisition process and do not describe the challenges. To the best of our knowledge, no method exists that focuses on the task of the extraction of production-relevant variables directly. However, advances exist in the employed techniques to find those production-relevant variables. Production-relevant variables are nothing less than outliers to a silent—not producing—smart factory.

Therefore, all outlier detection techniques, in general, may be used to detect production-relevant variables. The challenge is to project those variables in a way that outlier detection techniques can correctly classify them. Consequently, the focus lies on outlier detection (OD), especially on neural network (NN)-based unsupervised OD in CPSs in the Industry 4.0 domain. The focus lies on unsupervised techniques because a smart factory produces a significant amount of unlabeled data unsuitable for a supervised setting.

The section considers the latest advances in NN-based OD, in the security domain of CPSs, OD in the industry domain, transfer-learning for OD, ensemble-based OD methods and intelligent data pre-processing. Each area affects our proposed method.

### 3.1. Security Related Outlier Detection

Recent publications on machine learning in CPSs using recorded datasets and the work in OD often covers the security domain. The employed methods use OD for intrusion detection/attack detection and prevention. Kreimel et al. [12] achieve good results on a small dataset with a low feature space using k-nearest neighbor (k-NN) to differentiate outliers and a naïve Bayes classifier to predict the outlier class label. An advantage of Kreimel’s approach is that the approach can detect unknown cases. By separating detection and classification, training only known cases is computationally inexpensive for the naïve Bayes classifier. Bitton et al. [13] proposed a method based on principal component analysis (PCA), k-means clustering and a cluster-based local outlier factor (CBLOF) to reduce the feature space, form clusters and use CBLOF to classify abnormal clusters or outliers in the network’s traffic. Kim et al. [14] trained the expected behavior of a CPS with a long-short-term-memory (LSTM) neural network with an attention mechanism. The authors also use a running threshold that triggers if the 90th percentile is over or equal to 20 times the 20th percentile. Furthermore, the authors train a model per process or per univariate time series. However, their approach on a multivariate model does not show good results. In particular, each network recognizes specific characteristics of each process, and the authors use those NNs simultaneously to predict outliers.

We acknowledge the idea of a running threshold because a fixed threshold would lead to worse OD results over time. We further advance this technique in varying the lower bound and static upper bound for the AE ensemble. An et al. [15]. propose a mixture of k-means clustering and multivariate Gaussian models to first equally distribute feature space for the different models and then train each model independently to recognize specific characteristics of an artificial production bus system with multiple buses.

We follow the idea of equally distributed subsets of training data to learn different parts of complete systems. We advance the step by calculating the information loss to subscribe variables and receive unbiased/disturbance-free information. Yan et al. [16] introduce a feature generation step before training. First, univariate features will be enlarged, e.g., standard deviation, min, max and mean. Next, multivariate features are categorized into three groups, in which the first will apply, e.g., covariance, the residual of physical models with their mean error rates; in the end, learned features are added to the feature space of multivariate features. Learned features are the output of the middle hidden layer of an AE, which is smaller than its input feature vector. These new features are fed to an extreme learning machine (ELM), where a threshold helps differentiate outliers from standard data. Their approach is tested on artificial data to introduce attacks and on real-world gas turbines, where their approach sticks under a 1% rate of false alarms. The advanced pre-processing should be noted here, which could also be utilized in our approach in the future.

Bernieri et al. [17] compare different machine learning techniques to classify outliers and prevent attacks on a secure water treatment testbed. They evaluate support vector machines (SVM), random forests, and k-nearest neighbor (KNN) in a supervised setting. For unsupervised learning, they use one-class SVMs (OCSVM) and an AE with a fixed threshold to rate outliers. In general, supervised approaches outperform unsupervised techniques. However, the unsupervised methods could not be fully evaluated because of a flaw in the training data, so the found outliers could not always be directly validated as an outlier and wrongly taken as an error. OCSVM performs a little better than threshold-driven AEs. False data injection attacks are classified via an approach of Potluri et al. [18]. The authors fed data from a process control plant into an AE and a deep belief network (DBN) and used a downstream SVM or softmax regression (SMR) to assign a class to an outlier. Macas et al. [19] built a convolutional LSTM-based AE with an attention layer to classify outliers utilizing a threshold after a Bayesian Pearson correlation analysis on the secure water treatment data set. Kasongo et al. [20] use a deep feed-forward NN to classify attacks on two different data sets, with a threshold found during field trials. Lee et al. [21] use a combination of feature extraction using stacked AEs and linear SVM to recognize impersonation attacks in CPSs. Siegel et al. [22] prevent attacks on the secure water treatment using different NNs, feed-forward AEs, recurrent AEs and a 1-D convolutional NN. The authors compare results with isolation forts, minimum covariance determinant (MCD) and CBLOF.

Some approaches use an NN for pre-processing, whereas others use statistical or mathematical features to add to the feature proposed through the data set. Furthermore, machine learning techniques such as Bayesian classifiers, SVMs, k-NN and PCA are combined with simple deep feed-forward NNs, LSTMs or convolutional NNs to find outliers in CPS data. In pre-processing, only one publication uses statistical, mathematical and learned features for OD successfully. A fixed threshold is often used to identify outliers, while only one publication proposes a running threshold [14].

### 3.2. Industry-Related Outlier Detection

A few publications focus on OD for fault diagnosis in industry CPSs. Inoue et al. [23] used a combination of a derived-feature vector based on log lines to compute an outlier factor and an LSTM AE to learn the outlier factor’s representation. The authors use a threshold found during experiments to determine outliers from normal behavior. Eiteneur et al. [24] trained an LSTM-based NN on artificial data and explained the results mathematically. Additionally, the authors use the LSTM on a univariate power demand data set to validate their findings. Meng et al. [25] use a time convolutional AE (TCN-AE) to classify outliers. Savic et al. [26] train two competing AEs, trained offline, stripped to run on edge devices, to classify abnormal behavior in of CPSs in cellular networks. Ketonen et al. [27] facilitate a variational AE based on gated recurrent units (GRU) with static and dynamic time-based features to reason over outliers. Additionally, the authors rank the features in the reconstructed feature vector based on the contribution to the reconstruction error. Consequently, the influence of each feature on the outlier score can be shown. This influence can be used as a hint towards the origin of an outlier. Balogh et al. [28] train a constraint-programming model by computing additional features such as feature relationship costs. If a constraint is violated, the error can be visualized with the help of the model.

Both Ketonen et al. [27] and Balogh et al. [28] contribute to explainable AI models. The former enables the explainability by hints in the ranked feature vector, and the latter uses a visualizable model to show broken constraints. As the proposed approaches are based on simple datasets, the results are not directly transferable to a complex production line. However, both approaches propagate ideas for explainable faults, which is remarkable. In particular, the visualization of the contribution to the reconstruction error holds potential.

### 3.3. Transfer-Learning Outlier Detection

First progression has been made in using transfer learning in the area of Industry 4.0 to find outliers. Zhou et al. [29] describe paired CNNs, which the authors call a Siamese NN. The output of the hidden layer is an additional input of the second CNNs hidden layer, and as such, both CNNs are connected to each other. Now, via an advanced loss function, the NN minimizes the transformation loss, the encoding loss, and the prediction loss simultaneously. Therefore, the NN is able to find a better transformation strategy, encoding strategy and, in the end, better prediction, which supports the OD. Another approach by Cui et al. [30] learns the features and characteristics of time series data from one domain to develop a model that can distinguish data points from a different domain. This is enabled by intelligent pre-processing of the data and the mathematical alignment of both domains, so the search space is kept the same. Li et al. [31] use the trained ResNet-152 and re-trained the first and last layers for OD in a bearing data set. The authors proved that it is possible via transfer learning to achieve good results in another domain.

Transfer-learning is only in its beginning stages for the CPS domain. Nevertheless, the results by Li et al. [31] provide promising results with the reuse of already trained NNs such as ResNet-152. The use of already trained NNs could speed up our method further in the future.

### 3.4. Ensemble-Based Outlier Detection

Ensemble machine learning approaches are another way to detect outliers. In particular, the consensus mechanism is of interest as we also propose an unsupervised ensemble method. Arman et al. [32] present an ensemble OD method where multiple decision trees are trained on a subset of the data set to differentiate outliers in simulated data of a CPS network. For consensus, each tree is weighted with a certain confidence level, and the vote multiplied by the confidence level is averaged per class and vote. In the end, the vote backed by the majority is given back as a result. Hu et al. [33] used a stacked denoising AE (SDAE) to reduce the feature space and trained the classification and regression tree (CART) on top of the reduced feature space, and XGBoost as ensemble classifier is used to rate the outliers. Dutta et al. [34] employ a deep sparse AE (DSAE) to use the hidden layer output as an input feature for subsequent LSTM-based and feed-forward NNs, which are trained on subsets of the original data sets. Both exist in a hierarchy of level-0, which learns to reconstruct the DSAE output, and level-1, which learns the output of the level 0 NNs. The consensus is the averaged result of each separate trained level-1 model. Hoque et al. [35] find outliers in a maritime CPS. The authors use spectral clustering to gather route information for training. Two different LSTMs are trained, one for ships and one for route information. Therefore, outliers can be reasoned of the ship’s attributes become abnormal or its route information. There is no consensus, and each NN represents different aspects of the surveyed data. In contrast, Al-Abassi et al. [36] split training data in two balanced sets that are used to train two AEs. Those AEs are used for feature reduction, and the hidden layer’s output is used to train subsequent random forest (RF) classifiers. The results of these RF classifiers are fed to another RF classifier to distinguish between standard data and outliers. Therefore, the consensus is achieved via the separate RF. Feng et al. [37] combine one feed-forward NN and an LSTM NN to fed their hidden layer to another feed-forward NN to reconstruct the given value and at the same time do a one-time prediction in the future. Together with Baysian filtering, it is possible to calculate an anomaly score based on the predicted mean and covariance of the sensor value. Here the consensus is performed via a subsequent algorithm and a threshold over the anomaly score.

To summarize, ensemble methods in NN-based OD have significantly higher complexity of operation. Moreover, the consensus can be grouped into four categories: majority voting, specialized networks, averaging results and subsequent algorithmic evaluation. In majority voting, the majority decision and the greatest confidence declare the outlier, whereas only one network declares the outlier in specialized networks. In averaging results, the average of all used methods and a certain threshold classify an outlier. However, also a subsequent algorithmic evaluation such as an RF is possible to distinguish between outliers. We also favor the idea of voting because specialized networks can rate certain areas of a production line. In contrast, to the propagated methods, we use a minority voting of specialized AEs to identify outliers.

### 3.5. Intelligent Pre-Processing Data before Training

Two publications consider the NN-based pre-processing of training data and use different techniques for variable selection before training [38,39]. Cai et al. [38] propose a deep support vector description (SVDD) together with variational AEs (VAEs) to detect out-of-distribution values in training sets to reduce noise. Only non-distorted training data are fed to upstream machine learning models to have higher confidence in predictions. Tertytchny et al. [39] obtained higher accuracy and precision by pre-selecting only ranked class labels. Only disjoint features for each class are selected for training the machine learning models, and shared features with other classes are discarded.

On the one hand, the models learn significant features according to the class label and, on the other hand, spare feature spaces and speed up training. Although both methods [38,39] are not directly related to production-relevant variables, they are used to identify suitable variables for training or reduce feature space as we propose with our method. However, both methods [38,39] demand labeled data, which is insufficient in an Industry 4.0 smart factory environment.

## 4. Unsupervised Selection of Production-Relevant OPC UA Variables

**Figure 2:**
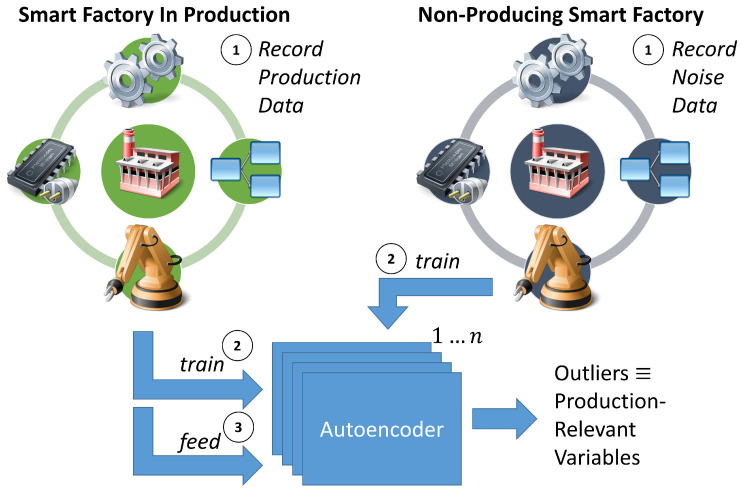
Overview flowchart of the training and selection process. (1) The smart factory is recorded twice: in production and non-producing. (2) The AEs are trained on the recordings. (3) Production data are fed to the AEs to detect production-relevant variables.

Our versatile approach is based on the simple idea of differentiating production activity from noise—invariable activity. The hypothesis is that active parts during production are more likely involved in any fault. The concept origins in the outlier detection community, in which autoencoder NNs learn the norm and identify outliers based on a set threshold. We exploit this concept in our approach (see Figure 2). Our approach consists of three major steps:

1. Record the smart factory for data;
2. Train AEs on recorded data;
3. Feed production data to detect production-relevant variables.

First, to differentiate production activity and noise, we record the same smart factory twice. One recording is conducted during production and comprises all available OPC UA variables. The other recording is performed on a non-producing smart factory to cover noise without any production activity. Next, autoencoders are trained on the recorded production and the noise-reflected datasets. Afterwards, the production data are fed to the trained autoencoders to distinguish between noise and active—production-relevant—variables. However, technical limitations hinder a straightforward implementation of the steps above—the challenge of transfer the maximum information with limited bandwidth and transfer capabilities. Furthermore, the challenge hinders the proper training of the AEs because the recording is highly affected.

The smart factory delivers large amounts of data if all variables are surveyed in parallel. As a result, the network becomes overloaded, and events are lost due to network latency. Those lost events have an impact on the quality of the recordings. Therefore, the information loss needs to be quantified. Now, the number of variables can be computed based on the quantified information loss that can be recorded in parallel. Next, the non-producing smart factory *n*-times to gather all noise. Each recording batch is used to train one of the *n* AEs. Therefore, *n* AEs cover the noise of the entire smart factory. Consequently, an AE ensemble exists that learned to reconstruct the noise of different parts of the smart factory. The analyst has *n* specialized AEs that rate new events. If only one AE is able to reconstruct the new event according to the model-specific threshold, the event is flagged as noise. We refer to this voting procedure as minority voting. Furthermore, production data are also flawed and encompasses outlying data, e.g., large text messages, which are hard to interpret and retain minor information. Consequently, one production AE is trained to filter those events out via a model-specific threshold.

After both procedures, filtering via the production AE and minority voting, the fed production data are rated via the reconstruction error. The more active a variable is, the higher the reconstruction error. Once more, we exploit that fact to extract a subset of variables based on the computed number of monitorable variables in a separated procedure. This procedure can be executed multiple times on the rated dataset to an adequate information loss—chosen by the analyst. Moreover, as a benefit, the confidence in subsequent recordings is higher as the possible information loss is now known, and only the most production-relevant variables are in focus. The following subsections will detail every step further.

### 4.1. Data Acquisition and Information Loss

Even currently, the retrieval of all available information is not possible due to network capacity and latency. Therefore, proper data acquisition for training the AEs is challenging. First, our approach is based on the idea that information loss is quantifiable in a smart factory. Information Loss is the degree of information becoming lost during subscription and retrieval, mainly caused by certain bottlenecks in the smart factory environment, e.g., the different communication buses or gateways. For example, the OPC UA gateways’ buffer has a capacity that if a vast number of variables are simultaneously subscribed, a certain amount of information are lost, even if the information was sent by the CPSs. Consequently, the communication buses are polluted, which leads to new faults [6]. Besides contextual faults, faults leading to production slow downs and production outages may appear. Slow downs may appear via discarded acknowledge events (exceeded round trip times in the bus system). Those events are sent after each process is completed. In order to proceed further, the events need to be resent. Even worse is a discarded command signal or the health check signal, which would stop the manufacturing process, leading to production outage. Such an outage could cause the production line to be in an unpredictable state.

We quantify the Information Lost (IL) as follows.

(1) $$\begin{array}{c} IL=\frac{Number\;of\;counter\;events\;received\;in\;time\;span\;t|all\;OPC\;UA\;variables\;subscribed}{Number\;of\;counter\;events\;received\;in\;time\;span\;t|counter\;variable\;subscribed\;only} \end{array}$$

To quantify the information loss, the analyst introduces a new OPC UA variable in the production system in one of the CPSs. The counter variable changes with a specific pattern, e.g., 15 Mhz, and increases an internal counter. This behavior provides a defined pattern for a subscription. Furthermore, the pattern allows recognizing missing, changed events. Moreover, the analyst can utilize this behavior to track the information loss in the smart factory. For this, the analyst subscribes to all OPC UA variables at once for a certain time span, e.g., one minute. Afterward, the counter-changed events in the log file are counted. This sum will be divided via the sum of counter-changed events if only the counter variable is subscribed for the same time span.

Now, the analyst is able to compute the number *N* of variables that can be subscribed with minor IL:

(2) $$\begin{array}{c} N_{subscribable\;variables}=IL\cdot \left|OPC\;UA\;Hierarchy\right| \end{array}$$

### 4.2. Autoencoder Ensemble Training and Utilization

In the ensemble, production data are filtered and outliers are removed through a set threshold based on the reconstruction error of a production trained AE (see Algorithm 1 Part 1). Production outliers are large messages that are hard to interpret and retain minor information. One example is a sent message encompassing an array that consists of the entire RFID data written to an RFID Tag. Another example is HTML that is sent through the network for the web interface of the central Management Station. The production AE is trained on a log file (pData) that is generated while recording multiple production cycles. For the production AE, all available variables are subscribed, although the IL is known. In our experiments, the recorded messages are enough to filter out production outliers based on the model-specific threshold. The threshold to consider events as production outliers is the 70th percentile of all MSEs rated by the production AE. We found this percentile during field trials.

Next, the AE ensemble consists of multiple noise-focused AEs that learn the behavior of non-production-relevant—noise—variables while the smart factory is not in production. For example, recordings can be performed during the first start of the production line while maintenance cycles or at the end of a production outage. *N* is now used to calculate *Z*, which is the number of AEs needed to cover the noise of the smart factory fine-grained(with minor IL).

(3) $$\begin{array}{c} \left\lceil \frac{\left|OPC\;UA\;Hierarchy\right|}{N}\right\rceil =Z_{number\;of\;AEs} \end{array}$$

With *N* and *Z* known, all variables are split equally in *N*-sized bins. Each subset of variables is subscribed and recorded for a certain time span, e.g., three minutes. The last subset will contain fewer variables. Therefore, a recording encompasses *N* variables maximum. These recordings are used to train the *Z* noise-focused AEs in the ensemble. Next, all events in the remaining pData are rated according to the following algorithm (see Algorithm 1 Part 2).
**Algorithm 1** Minority-Voting through the AE-Ensemble for the Detection of Production-Relevant Variables**Input:** Production Data (*pData*), AE Ensemble (*pAE*, *Z* = [noise-focused AEs]),*toVote* = table(columns = [‘relevant’, ‘mse’])***% Convert data to a numerical format.***$conv_{pData}=convert\left(pData\right)$$pred_{conv}=pAE.predict\left(conv_{pData}\right)$$pmses=calculateMSE(conv_{pData},pred_{conv})$***% Part 1: Remove all production outliers from subscription data.******% Border is the 70 percentile of the production AE MSEs (upper bound).***$toVote_{conv}=conv_{pData}\left[\left(conv_{pData}<pmses.percentile\left(70\right)\right)\right]$***% Part 2: Rate the remaining events by minority voting and save the lowest MSEs***$While\;ae_{z}=Z.hasNext\left(\right)\;do$$pred_{toVote}=ae_{z}.predict\left(toVote_{conv}\right)$$nMSEs_{z}=calculateMSE\left(toVote_{conv},pred_{toVote}\right)$***% AE threshold is the 30 percentile of the MSEs of the non-working smart factory after******% training (varying bound). If the current MSE (working smart factory) is minor, the******% lower bound of non-working smart factory AE the value is clearly not******% production-relevant.***$toVote_{conv}\left[‘relevant’\right]=False\;if\;nMSEs_{z}<ae.threshold$***% Save lowest noise-focused MSEs overall AEs***$toVote_{conv}\left[‘mse’\right]=\left\{\begin{array}{c} nMSEs_{z},toVote_{conv}\left[‘mse’\right]\;is\;None\\ nMSEs_{z}\left[\left(nMSEs_{z}<toVote_{conv}\left[‘mse’\right]\right)\right],otherwise \end{array}\right.$Endwhile$Return\;voted_{pData}=toVote_{conv}$

**Figure 3:**
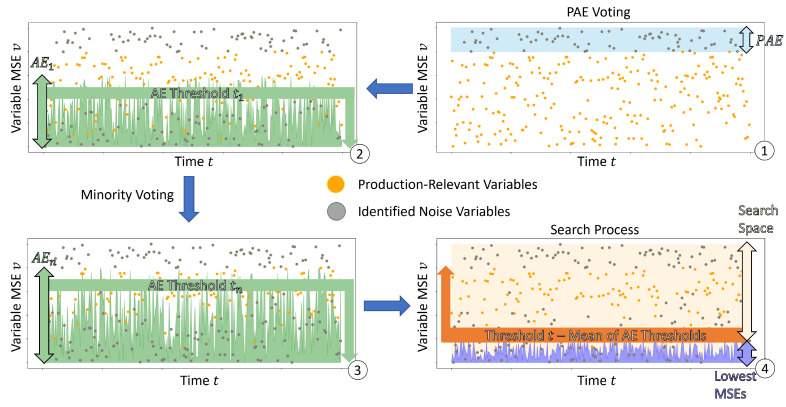
Illustration of AE-Ensemble Utilization. (1) Production AE (PAE) removes outliers from production data to clean the pData. (2–3) Each AE reconstructs the events encompassed in pData differently and flags events as noise (minority voting). (4) The search process starts from the mean of all AE Thresholds and increases until only the computed *N* production-relevant variables are left.

Each AE reconstructs the entire pData and classifies each event under a model-specific threshold as noise—or production-relevant false. The more active a variable is, the more different the log pattern and the higher the reconstruction error. The model-specific threshold is the 30th percentile of the MSEs during training. Consequently, each AE has a unique threshold, and we refer to this as the varying lower bound, whereas we refer to the production AE threshold as a static upper bound. The boundaries define the search space where production-relevant variables exploration takes place. Figure 3 illustrates the process. After the production AE filtering and the minority voting has taken place, every event has the lowest MSE assigned. The iterative search process, which can be performed multiple times on an already voted dataset, is executed as follows.

(4) $$\begin{array}{c} voted_{pData}\left[\left(voted_{pData}{[}^{\prime }relevant^{\prime }]\right)\&\left(voted_{pData}{[}^{\prime }mse^{\prime }]>t\right)\right]|t=Z.meanAEthreshold+x \end{array}$$

Here, *x* is raised by 0.02 until condition $N==|unique(voted_{pData})|$ is met. Unique returns the set of unique variables in $voted_{pData}$. The analyst is able to adjust the IL by a reasonable value, so a subsequent analysis is not affected. For some analyses, the minimum of IL is necessary, whereas, for others, 10% or 20% IL is also acceptable. As a result, *N* reasonably increased. The method will return the *N* most active variables during production, which additionally will be more likely involved in any faults.

## 5. Experiments

Before we discuss the results (Section 5.3), we describe our state-of-the-art production-equal smart factory at the Darmstadt University of Applied Sciences (DUAS) (Section 5.1), the datatypes, the conversion strategy and the used AE architecture (Section 5.2).

### 5.1. Testbed

**Figure 4:**
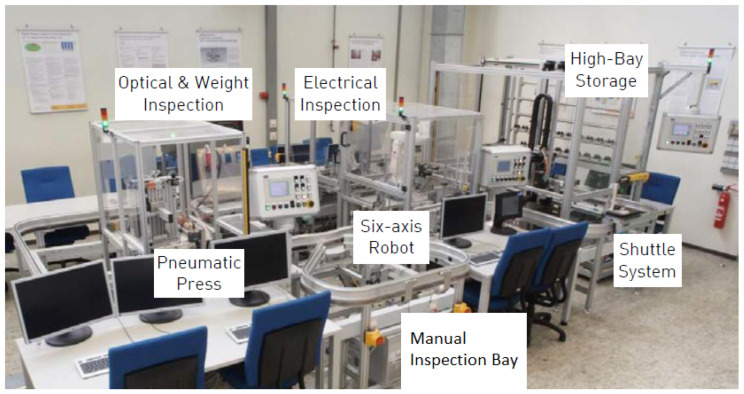
Production-equal smart factory at the DUAS [6]. A monorail shuttle system connects the high-bay storage with a six-axis assembly robot, a press, the optical and weight inspection and the electrical inspection station. Unassembled products will be delivered clockwise via the stations. In case of a successful assembly, inspection stations successfully proofed the relay; the assembled relay will be stored back into the high-bay storage. Reprint with permission from [6], “CONTEXT: An Industry 4.0 Dataset of Contextual Faults in a Smart Factory”; published by Elsevier, 2021.

At the DUAS, the smart factory produces electrical relays in full automation (Figure 4). Unassembled parts are stored in a high-bay storage by a three-axis robot. The three-axis robot deploys the parts based on a pallet onto a shuttle in a monorail shuttle system. The shuttle system interconnects all stations. Next, a six-axis robot assembles the relay. Afterwards, the press squeezes the relay to assure connectivity between the parts. Subsequently, the relay is checked by the optical and weight inspection station as well as the electrical inspection. The relay will be sorted out if the proof routine detects a faulty relay; otherwise, the three-axis robot at the high-bay storage stores the relay back in one of the empty slots, ending a production cycle.

The presented method is intended to speed up problem-solving by focusing the analysis on production-relevant variables. We published the CONTEXT dataset, which contains 16 production cycles as ground truth. In subsequent runs, contextual faults were introduced, where the context of multiple stations is necessary to determine the reason for the fault [6].

We identified three contextual faults [6] in the smart factory:

- Missing Parts. During high-speed production or a misplacement on the pallet, a part could fall off the pallet. Moreover, a not fully equipped pallet may be stored in the high-bay storage by accident. If a part is missing on the pallet, the employed automation will keep assembling without further notice. Moreover, the electrical inspection will detect the fault and sort out any faulty relays. However, each faulty relay is treated the same and not used for production anymore. If the fault of a missing part would be detected, the amount of waste could be reduced, and the parts could be reused. The contextual fault encompasses the high-bay storage, the assembly robot, the press and electrical inspection.
- Missing Pressure. The shuttle system and two stations in the production line work with pressure, the assembly robot and the electrical inspection. If the pressure starts diminishing, the production line slows down via the slower movement of the stations, and in the worst case, a downtime occurs. The contextual faults span the three subsystems.
- Shuttle Dropout. A shuttle dropout appears if the shuttle stays in the manual inspection bay. Both shuttles send positive feedback, but the production output is cut in half. Only the time between assemblies will vary. The contextual fault encompasses each employed CPS. Here, the contextual fault spans all involved CPSs.

These errors are used for validation. A domain expert evaluates the variables found with the method to determine how valuable they are for later analysis if one of the contextual error occurs.

### 5.2. Data, Transformation, and Training

The smart factory (33,089 OPC UA Variables [6]) was recorded twice for the presented approach. The variables encompass CPS, IIoT and environment information. One recording encompasses an active production process and the other a silent—non-producing—smart factory. Each recording was stored in a log file. The format and example of each data type is shown in Table 1.

Each row represents a change event. Furthermore, each event has a date, a nodeid, the changed value and the dtype (data type). The nodeid represents the path in the OPC UA Hierarchy, from root (namespace ns), over the corresponding station, different hardware or software modules to the variable name. The modules are dot-concatenated. The example shows a variable from the optical and weight inspection station (Station 20) and refers to the software routine SAP_Fehlercodes_PCo_iDB and the Start variable. The value was set to False, and the data type is Boolean. Currently, 13 different data types exist in the smart factory (see Table 2).

**Figure 5:**
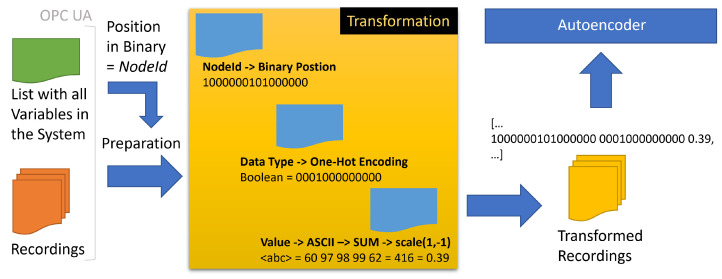
The transformation process. Numerical transformation of recorded events.

A transformation strategy should treat all data types and values equally, regardless of the data type. We choose a simple strategy to encode each event numerically for the AE training (see Figure 5). First, the *nodeid* is transformed into a binary representation. For that, the position of the unique variable in the list of 33,089 variables is transcoded into binary (e.g., position 33,089 > 1000000101000000). The transformation saves feature space in contrast to an otherwise one-hot encoding of the categorical values. Next, each value is transformed to the sum of its ASCII representation. Lastly, the data types are one-hot encoded. The *date* column is only used to sort the events and is pruned afterwards. Additionally, each *value* is scaled between −1 and 1 to reduce the distance between the categorical transformed *nodeid* and the one-hot encoded datatypes.

**Figure 6:**
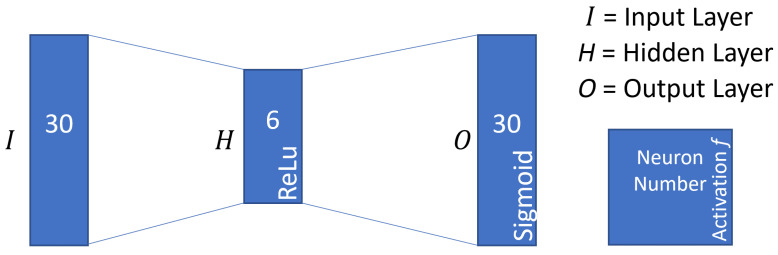
AE configuration.

The input vector of the AEs consists of 16 neurons for the *nodeid*, 13 neurons for the one-hot encoded data types, and one neuron for the value. Figure 6 provides an overview of the AE configuration. We employ simple dense AEs with a 30-6-30 configuration and rectified linear units (ReLu) and sigmoid as activation functions for fast training. The AEs are trained with a batch size of 10 encoded events and for 1000 epochs.

The measured IL is ~0.02215 in the production line that translates to ~732 subscribable OPC UA variables (*N*); Consequently, *Z* is ~45. We record each subset of the 732 variables for three minutes each. The last batch consists of 881 variables. As a result, the noise-recording took in total 135 minutes. The active production recording (pData) as published in [6] is our ground truth, with 16 successful assemblies and 35 min of production activity. After training, the AE ensemble votes the events in pData. For this, pData is transformed into numerical data; the production AE filters out production outliers, and the noise-focused AEs rate the events. The more active a variable is, the more uncommon its pattern and the higher is the MSE. The search procedure starts from *Z.meanOfAEthresholds* and increased until the chosen number of variables is selected. Here, we increased the IL to ~0.03, which translates to ~1063 chosen OPC UA Variables. The increase in IL was performed after a consultation with the domain expert.

### 5.3. Results

**Figure 7:**
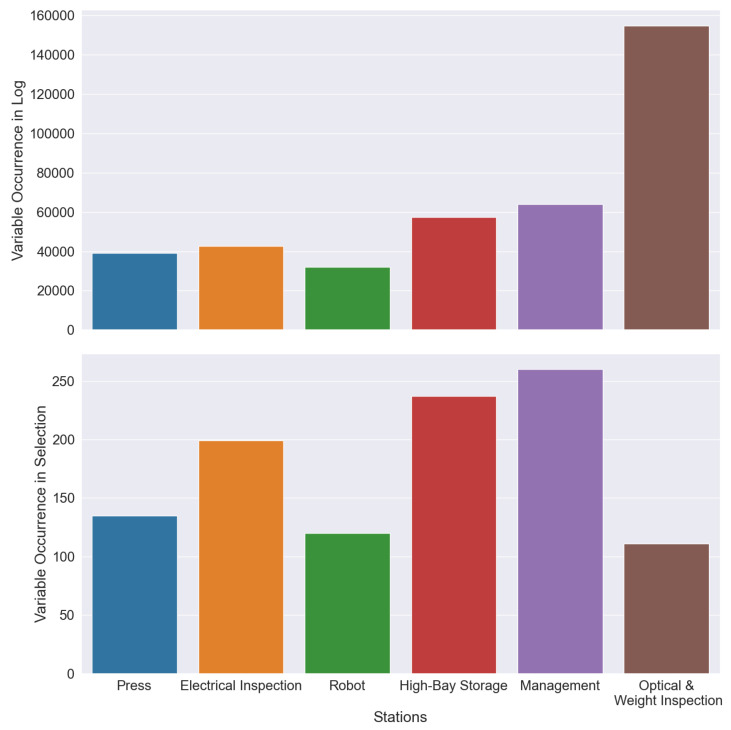
Number of OPC UA Variables before selection in pData (**top**) and after selection (**bottom**).

**Figure 8:**
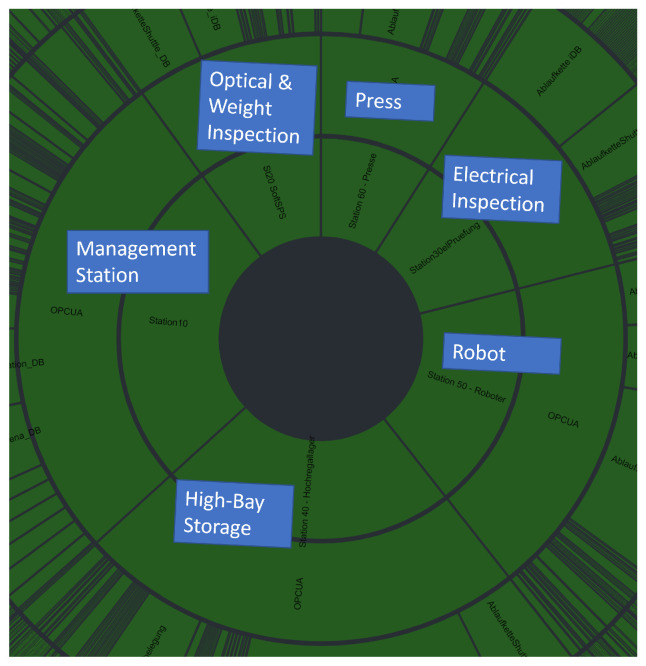
OPC UA Hierarchy (33,089 Variables) visualized in a sunburst chart. The more variables that exist in a subtree, the larger the section.

**Figure 9:**
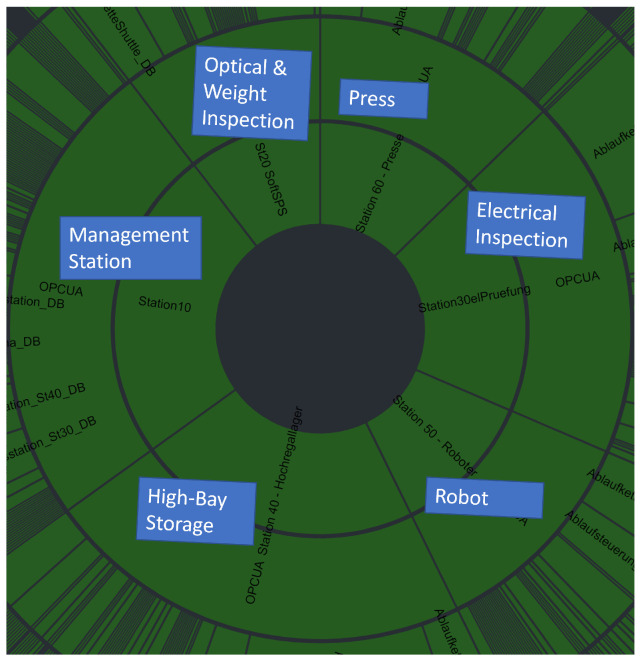
The selection result of 1063 OPC UA Variables visualized in a sunburst chart. The more variables that exist in a subtree, the larger the section.

The results, 1063 chosen OPC UA variables, are shown in figures (Figure 7 and Figure 9). Figure 8 provides an overview of the complete OPC UA Hierarchy for comparison to Figure 9. Figure 7 compares the number of variables before and after the selection was performed on *pData*. In *pData* (Figure 7, top with 389,990 value-changed events), the *Optical & Weight Inspection* is over-represented with >140,000 value-changed events. Furthermore, the *Management* station and the *High-Bay Storage* have around 80,000 value-changed events. Additionally, the *Press* and the *Electrical Inspection* have the same level of value-changed events (40,000 events). The *Robot* has the smallest amount of all recorded changed-value events (>30,000 events). In *pData*, value-changed events are not equal to the number of recorded OPC UA variables because some OPC UA variables (e.g., the axes of the Robot) will drastically change multiple times during production. Each change emits a value-changed event. The production data set encompasses 33,059 OPC UA variables. Therefore, the recorded production data set has a 99.9% coverage of all smart factory variables (33,089 variables). Consequently, recorded production data (*pData*) are representative.

After the selection procedure, the result set of 1063 variables becomes differently weighted (Figure 7, bottom). First, the finding is that the result set is in line with the expectations. The *Management* station, which is involved in every production step, is selected more often (>250 OPC UA variables). The *Management* station sends production instructions and receives production status updates. The *High-Bay Storage* and the *Electrical Inspection* have been selected more frequently (>200 OPC UA variables). The *High-Bay storage* is involved in every production cycle twice (deployment of materials, storage of factored relays). Therefore, the amount of OPC UA variables doubled, which is reasonable. The *Electrical Inspection* has multiple proof routines for different relays. As a result, the chosen variables have the second highest frequency. The *Press* and the *Robot* are selected as approximately equal. For instance, the *Robot* and the *Press* employ the same routine for every relay every time, which is reflected by the equal amount of chosen variables. Ergo, the frequencies of both stations are reasonable. Besides, for the *Optical & Weight Inspection*, the result set mirrors the frequencies of *pData*. The *Optical & Weight Inspection* has a lot of ground noise, where the components send massive value-changed events. The emission happens for two reasons. *Optical & Weight Inspection* is currently under active development, and variables are emitted more frequently for debugging. The other reason is that many proof routines malfunction through active development and send erroneous feedback to the *Management* station after every production instruction. As a side-effect of the development process, the active or influential OPC UA variables during production are less, than the variables of the Press and the Robot station. Therefore, the frequency of the chosen OPC UA variables of the *Optical & Weight Inspection* station is also reasonable.

Figure 8 visualizes the proportion of variables in the OPC UA hierarchy under each CPS in a sunburst chart. The larger the section is, the more variables are contained its subtree. The sunburst chart allows a direct comparison between all variables (33,059 variables, Figure 8) and the result set (1063 variables, Figure 9). In Figure 8, the *Management* station has most variables, followed by the *High-Bay Storage*. The *Robot* has more variables than the *Electrical Inspection*. *Optical & Weight Inspection* has fewer variables than the Press. An important finding is that the presented approach retains the proportions of the OPC UA hierarchy during the selection process. The OPC UA hierarchy is approximately equally downsized from 33,059 variables to 1063 variables. The AE ensemble automatically performs the reflection during minority voting without explicitly specifying the behavior during training. This is significant as we train our AEs on continuous subsets of all OPC UA variables rather than setting an AE for specific CPS. We can also show that over-represented variables (Figure 7, top) do not influence our approach as the number of selected variables of the *Optical & Weight inspection* CPS (Figure 7 (bottom) and Figure 9) is reduced to the approximately same percentage as represented in the OPC UA hierarchy (Figure 8). Moreover, Figure 9 is also an overview of the share of a CPS on the production process available via the production-relevant variable selection.

In the end, after a review of the result set, a domain expert verified that the found production-relevant variables hold information that enables the detection of the contextual faults proposed in our previous publication [6]. In an experiment, we were additionally able to gather around 60% more value-changed events during a production process with the selected variables. Moreover, the aspect is surprising but can be explained by another aspect of the selected variables. Most selected variables are simple types, e.g., boolean values or numbers, which in return have a smaller package size during transmission. Consequently, more information can be transmitted at the same time yield to an increase in throughput. An increase in throughput may be beneficial for every subsequent fault analysis.

## 6. Conclusions

In this publication, we presented an unsupervised approach to select production-relevant OPC UA variables. We were able to downsize an OPC UA hierarchy with 33,089 variables to 1063 variables. The downsized excerpt allows for a dense focus on production-relevant variables that are applicable, e.g., to reason over contextual faults [6]. The presented method is robust against over-represented variables in the fed production dataset and, at the same time, retains the proportions of the entire OPC UA hierarchy. By focusing on production-relevant variables, which respect the information loss, the network delivers the maximum information without interfering with the production process. As a result, the proposed approach contributes to the challenge of bandwidth, transfer capabilities and available information in Industry 4.0. Now, OPC UA variables of the smart factory can be surveyed in a fine-grained manner, which supports a subsequent context-aware diagnosis. With this work, we detail the data section of the TAOISM model [2,3] further and add an unsupervised method to select production-relevant variables automatically.

The unsupervised method employs an AE ensemble consisting of a production AE and multiple noise-focused AEs. The information loss in the production line is quantified. The higher the information loss, the more noise-focused AEs are needed to cover the noise of the entire factory. A minority voting procedure is presented. The production AE in the ensemble is needed to pre-filter the production dataset to remove production outliers and improve the downstream minority voting quality. In the method, recording, training, voting and selecting are separated. The selection procedure can be executed multiple times in real-time (<1 ms) based on an already voted production dataset to gather production-relevant variables to a known information loss. Therefore, the analyst can balance the production-relevant variables and the information loss to favor any algorithm in subsequent analyses.

Some limitations exist that should be named. The first limitation is the training time of AEs. We choose a simple dense-layered AE architecture to be quick in training, but for each AE, we need three minutes of noise recording, which sum up in an advanced smart factory with more CPSs. Here, a study is planned to distinguish the lowest possible subscription time to gather sufficient data for training. Furthermore, the entire OPC hierarchy is subscribed for the production dataset, which may have consequences such as lost command signals and production slowdowns. Therefore, the production data may have to be recorded multiple times with multiple fault-free production cycles. Next, the currently employed AEs are not learning time-dependent features and sequences, which may affect results. In the end, we only performed a qualitative evaluation of our results, which only one domain expert verified. A study is planned to elaborate our approach on our already recorded contextual faults quantitatively.

Currently, we are working on different improvements. In experiments, we add another AE to encode the features and use the output of its hidden layer (learned features) to feed the method. With the reduction in feature space, the proposed method can be used on a much broader feature space without enlarging the computation time. Furthermore, a study is planned to use already pre-trained neural networks. The amount of training data and the recording time may be reduced via few-shot training while maintaining the appropriate results. In the end, we plan to evaluate different AE architectures in terms of training time, computational efficiency and purposeful results.

We can perform real-time surveillance of the smart factory and collect fine-grained information to reason for upcoming contextual faults via the proposed approach. With this approach, we add another contribution to context-aware fault diagnosis, which supports personnel in identifying faults in a changing Industry 4.0 environment.

## Figures and Tables

**Table 1:** OPC UA Log File Format [6]. Reprint with permission from [6], “CONTEXT: An Industry 4.0 Dataset of Contextual Faults in a Smart Factory”; published by Elsevier, 2021.

Columns	Data Type	Example Values
date	DateTime	2020/07/01 16:15:40:647
nodeid	String	“ns=6;s=Station 20 OpenC.St20 SoftSPS.SAP_Fehlercodes_PCo_iDB .Start”
value	Values for Data Types (Table 2)	False
dtype	Data Types (Table 2)	Boolean

**Table 2:** Datatypes of all available 33,089 OPC UA Variables with Examples [6]. Reprint with permission from [6], “CONTEXT: An Industry 4.0 Dataset of Contextual Faults in a Smart Factory”; published by Elsevier, 2021.

Data Type	Node Count	Example Values
Boolean	17,874	True, “[False, ]”
Byte	4918	11
ByteString	406	b‘\xff...’
DateTime	5	2020-07-09 16:05:11.795000
Double	1	0.0
Float	1248	3.1233999729156494
Int16	3003	4
Int32	1822	16
Int64	6	2103635700381566
SByte	34	“[32, 32,...]”
String	93	V3.0, “[‘+40,,’, ”, ”, ”, ”]”
UInt16	2655	2
UInt32	1024	3
